# Microbiota, Epithelium, Inflammation, and TGF-β Signaling: An Intricate Interaction in Oncogenesis

**DOI:** 10.3389/fmicb.2018.01353

**Published:** 2018-06-26

**Authors:** Xin Pang, Ya-jie Tang, Xiao-hua Ren, Qian-ming Chen, Ya-ling Tang, Xin-hua Liang

**Affiliations:** ^1^State Key Laboratory of Oral Diseases, West China Hospital of Stomatology, Sichuan University, Chengdu, China; ^2^National Clinical Research Center for Oral Diseases, West China Hospital of Stomatology, Sichuan University, Chengdu, China; ^3^Department of Oral and Maxillofacial Surgery, West China Hospital of Stomatology, Sichuan University, Chengdu, China; ^4^Key Laboratory of Fermentation Engineering (Ministry of Education), Hubei Provincial Cooperative Innovation Center of Industrial Fermentation, Hubei Key Laboratory of Industrial Microbiology, Hubei University of Technology, Wuhan, China; ^5^Department of Stomatology, Sichuan Academy of Medical Sciences & Sichuan Provincial People’s Hospital, Chengdu, China

**Keywords:** microbiota, colorectal cancer, TGF-β signaling, epithelial barrier, immunity

## Abstract

Microbiota has been widely considered to play a critical role in human carcinogenesis. Recent evidence demonstrated that microbiota, epithelial barrier and inflammation has made up a tightly interdependent triangle during the process of carcinogenesis. Hence, we discussed the triangle relationship of microbiota dysbiosis, epithelial barrier dysfunction and dysregulated immune responses to elucidate the mechanisms by which microbiota induces carcinogenesis, especially highlighting the reciprocal crosstalk between transforming growth factor-β signaling and every side of the tumorigenic triangle. This sophisticated interaction will provide insight into the basic mechanisms of carcinogenesis and may bring new hope to cancer prevention and therapeutic intervention.

## Introduction

The microorganism is now seen as a “new organ” in human. The “new organ,” which contains an enormous number of cells and genetic material, inhabits in body surfaces and cavities connecting with exterior in physiological conditions (Marchesi et al., 2016; Zitvogel et al., 2016). One of the characteristics of microbiota is its individuality as it is shaped by genetics, life and dietary styles, medication use and microbial exposure of hosts (Zitvogel et al., 2016). The complex community of microorganisms acts as an integrated ecosystem to provide benefits with healthy hosts. On the contrary, deviating from microbiota equilibrium has been regarded as an important factor in diseases intertwined with gene and environment (Bultman, 2014).

The suspicion that microbiota imbalance is involved in the course of carcinogenesis has been in the Spot-LIGHT in the past few decades. Cancers driven by infectious agents were estimated to comprise of 15–20% of the global cancer burden in 2012, among which *Helicobacter pylori* (*H. pylori*), human papilloma viruses (HPV), hepatitis B (HBV) and C viruses (HCV), and Epstein-Barr virus matter a lot (IARC Working Group on the Evaluation of Carcinogenic Risks to Humans, 2012; Bhatt et al., 2017). Microbiota dysbiosis refers to microbial maladaptation or imbalance of quantity and quality, especially imbalance in microbial richness and function, which makes commensal bacterium become pathogenic bacterium in host with genetical defect or dysregulated inflammation (Bhatt et al., 2017). It is reported that dysbiosis is associated with a large amount of malignancies and is implicated in the formation of cancers such as colorectal cancer (CRC) (Schwabe and Jobin, 2013; Perez-Chanona and Trinchieri, 2016). Accumulating evidence showed that the mechanisms of microbiota dysbiosis-driving oncogenesis may include: (I) integrating oncogenes of oncovirus into host genomes or compromising genomic instability, (II) triggering cancer by promoting inflammation and dampening immune-surveillance of transformed cells, (III) subverting the integrity of epithelial barrier, (IV) contributing to the resistance to cell death and disturbing signaling pathways related to carcinogenesis. Intriguingly, this indicated that microbiota, epithelial barrier and inflammation make up a tightly interdependent triangle during the process of carcinogenesis driven by microbiota (Fox and Wang, 2007; Couturier-Maillard et al., 2013; Hu et al., 2013; Francescone et al., 2014; Ojesina et al., 2014). However, the molecular mechanisms of the interaction of microbial dysbiosis, barrier failure and dysregulated inflammation regulating carcinogenesis have remained unclear (Schwabe and Jobin, 2013; Garrett, 2015).

Transforming growth factor-β (TGF-β) signaling pathway acts as a tumor-suppressor in normal epithelium or early stage of oncogenesis. However, like a coin with two sides, tumor-promoter role of TGF-β comes along that facilitates cell proliferation and immune-evasion (Zhang et al., 2006; Pardali and Moustakas, 2007; White et al., 2010). Mechanistically, TGF-β maintains homeostasis and blocks tumor formation through enhancing cell cycle arrest and apoptosis via canonical TGF-β-SMADs pathways. As TGFβR on tumor cells mutated, TGF-β accumulated in extracellular matrix, shaping a tumor friendly microenvironment that favoring proliferation and invasion of tumor cells. And TGF-β expression was correlated with angiogenesis, myofibroblast formation and inflammatory cells recruitment via non-canonical PI3K/Akt, the nuclear factor κ light-chain-enhancer of activated B cells (NF-κB), or ERK signaling (Iglesias et al., 2000; Lu et al., 2006; Bian et al., 2009; Freudlsperger et al., 2013). Notably, TGF-β realigns immune cells and abrogates the function of tumor-suppressing immune cells, such as dendritic cells (DCs) and T effector cells, to help tumor cells escape from immune-surveillance (Yang, 2010). Intriguingly, plenty of evidence showed that the mechanism of TGF-β signaling in favor of tumorigenesis also correlates with dysregulated inflammation microenvironment driven by microbiota.

In this review, we will outline current understanding of the mechanisms by which microbiota induces cancer and the impact of TGF-β signaling pathway in carcinogenesis driven by microbiota. As we learn more about the intricate relationship between microbiota and cancer, it may help indicate therapeutic targets and biomarkers of cancer (Zeller et al., 2014).

## Microbiota-Epithelial Interaction and Its Interference by TGF-β Signaling

The well-maintained mucosal barrier in gut or oral cavity, consisted of the mucous layer, the stratum corneum, immune cells, secretory immunoglobulin A (sIgA) and antibacterial peptides, is a multi-level defender to separate human tissues from microbiota (Schwabe and Jobin, 2013). Anatomic disruption, microbial composition and mucus production changes can bring about epithelial barrier dysfunction and microenvironment disruption (Schwabe and Jobin, 2013). They grant pathogens and commensal microorganisms access to the epithelium, and then their metabolites, toxins, as well as proteins may regulate tumorigenesis. Intestinal microbiota dysbiosis and its interaction with a functionally impaired mucosal barrier lead to ulcerative colitis. In up to 30% of affected patients after 35 years of ulcerative colitis has the occurrence of CRC (Rogler, 2014). Mucins, the highly glycosylated proteins, were the major component of the mucus that protected underlying epithelium. Absence of mucins increased proliferation and migration, as well as decreased apoptosis of intestinal epithelial cells (IECs). Muc2-knockout mice frequently developed into intestine adenomas and gradually progressed to CRC (Velcich et al., 2002). Bacterial translocation referred to the migration of bacteria and bacterial products to mesenteric lymph nodes or extra-intestinal sites from the gut. Increased translocation of intestinal bacteria contributed to hepatic inflammation and fibrosis, as well as promoted hepatocellular carcinoma (HCC) (Wiest and Garcia-Tsao, 2005; Dapito et al., 2012). While gut sterilization by antibiotic management could reduce HCC in the late stage of hepatocarcinogenesis of mice (Dapito et al., 2012). These indicated that destruction of host/flora equilibrium of epithelial barrier was a critical step in microbial associated diseases, including cancer (Sellers and Morton, 2014; Taur and Pamer, 2016).

Microbiota not only provides nutrients and helps to abstract calories for the hosts, but also produces detrimental metabolites favoring noxious inflammation and tumor formation (Bhatt et al., 2017). Evidence is emerging that sulfides, nitrosamines, hydrogen peroxide and deoxycholic acid (DCA) are the main detrimental metabolites of microbes related to carcinogenesis. Some *Firmicutes* and *Bacteroides* sp. ferment excessive protein of the host into sulfides and nitrosamines, which may induce DNA alkylation and mutations in host cells. Glycosulfatase in *Bacteroides* could catalyze sulfomucins to release sulfides, contributing to mucins degradation and carcinogenesis (Carbonero et al., 2012; Bhatt et al., 2017). And *Enterococcus faecalis* can produce extracellular superoxide, which also produced by host cells in inflammatory conditions, spontaneously deriving hydrogen peroxide in colon (Huycke et al., 1996, 2002). Hydrogen peroxide can diffuse into epithelial cells passively and form hydroxyl radical which leads to DNA double-strand break, base modification and DNA-protein cross-linking by iron-catalyzed reactions (Huycke and Gaskins, 2004; Wang et al., 2008; Sears and Garrett, 2014). *Clostridium scindens* converts primary bile acids in colon into secondary deoxycholic acid such as DCA. DCA perturbates cell membranes and releases arachidonic acid, which converted to prostaglandins and reactive oxygen species (ROS), exacerbating DNA damage and initiating carcinogenesis (Ridlon et al., 2016). Hydrogen sulfide and acetaldehyde are not inert bystanders too, resulting in inflammation and genomic instability, and promoting CRC development (Huycke and Gaskins, 2004; Windey et al., 2012; Schwabe and Jobin, 2013). Thus, intestinal bacteria have the potential to stimulate excessive proteins to form detrimental metabolites, genotoxins and tumor-promoters, through DNA double-strand breaks and activation of the DNA damage (**Table 1**).

**Table 1:** Oncometabolites from bacteria leading to noxious inflammation and tumor formation.

Oncometabolites	Related microbes	Effect	Reference
sulfides	*Firmicutes Bacteroides*	DNA alkylation and mutations;	Carbonero et al., 2012;Bhatt et al., 2017
Nitrosamines	*Firmicutes Bacteroides*	DNA alkylation and mutations;	Carbonero et al., 2012;Bhatt et al., 2017
Extracellular superoxide	*Enterococcus faecalis*	Indirect DNA double-strand break, base modification and DNA-protein cross-linking;	Huycke et al., 2002;Wang et al., 2008
Secondary bile acids	*Clostridium*	Indirect DNA damage; mitogen;	Yoshimoto et al., 2013;Ridlon et al., 2016
Acetaldehyde	*Streptococcus Neisseria Candida*	DNA damage and impaired DNA repair;	Hooper et al., 2009
Butyrate	*Clostridia Firmicutes*	Anti-proliferation and inflammation; pro-expression of p53;	Garrett, 2015;Vipperla and O’Keefe, 2016
ROS	*Clostridium Bacteroides fragilis* HCV *Helicobacter pylori*	DNA damage and mutations;	Obst et al., 2000; Goodwin et al., 2011; Chusri et al., 2016; Ridlon et al., 2016


Evidence supports the role of bacterial derived toxins in driving cell transformation mainly by their genotoxicity and interference with host-derived signaling pathways (Bhatt et al., 2017). Colibactin is the product of “*pks*,” a genomic island of *Escherichia coli* strains of phylogenetic group B2. Infection of eukaryotic cells with *pks*^+^ *E. coli* induces DNA double-strand break and activation of DNA damage signaling cascade, resulting in breakage–fusion–bridge cycles and the increase of anchorage-independent growth, which contribute to CRC development (Nougayrede et al., 2006; Cuevas-Ramos et al., 2010). Arthur et al. (2012) demonstrated that azoxymethane (AOM)-treated Interleukin-10 (IL-10) knockout mice developed invasive carcinoma in mono-colonized with the *pks*^+^ *E. coli*. On the contrary, AOM-treated IL-10 knockout mice mono-colonized with *E. coli* depleted *pks* showed declined tumor multiplicity and invasion (Arthur et al., 2012). Cytolethal distending toxin (CDT) derived from Gram-negative bacteria is also genotoxic factor that has intrinsic DNase activity. Nuclease CdtB travels to host cells with the help of CdtA and CdtC subunits, where it creates DNA lesions and results in intestinal hyperplasia of mice (Nesic et al., 2004; Shen et al., 2009). *Bacteroides fragilis* toxin (BFT) is a virulent factor that damages host DNA by eliciting ROS production, and is closely associated with signal transducer and activator of transcription-3 (STAT3)- and T helper 17 (Th17)-dependent inflammation and CRC (Wu et al., 2009; Garrett, 2015). Furthermore, Goodwin et al. (2011) identified that BFT upregulated the expression of spermine oxidase in colonic epithelial cells which catalyzed polyamine catabolism and ROS production, resulting in CRC formation (Goodwin et al., 2011). Thereby, a diverse array of virulence factors of bacterium and their pathways may manipulate basic host cell functions, such as proliferation and invasion, to contribute to carcinogenesis.

Recently, an association between virulence factors and host pathways in the course of carcinogenesis has been recognized, especially protein toxins. FadA encoded by *Fusobacterium nucleatum* adheres to lectins and E-cadherin on the surface of epithelial cells (Rubinstein et al., 2013). Similarly, *H. pylori* bears *cagA* that encodes CagA, an antigenic effector protein interacting with E-cadherin (Abreu and Peek, 2014). The competitive binding of FadA/CagA and E-cadherin impairs the complex between E-cadherin and β-catenin, leading to the activation of β-catenin signaling, which regulates downstream genes, such as c-MYC, *cdx1* and *Ccnd1* to promote cells proliferation of CRC (Murata-Kamiya et al., 2007; Rubinstein et al., 2013). Besides, AvrA secreted by gall bladder cancer associated *Salmonella*, has also been suggested to activate β-catenin signaling by suppressing β-catenin ubiquitination and increasing its phosphorylation. β-catenin activated by AvrA increased the expression of cyclin D1 and matrix metalloproteinase (MMP)-7, promoting colonic carcinogenesis (Samaras et al., 2010; Lu et al., 2014). Hence, protein toxins may play an important role in enhancing transformation of epithelial cells by affecting genomic stability and proliferative signaling.

Transforming growth factor-β signaling has been shown to play an important role in microbiota-epithelial interaction. Firstly, special detrimental metabolites increased TGF-β expression. *Clostridium* exacerbated the production of TGF-β by IECs, possibly through its metabolites, such as butyrate, acetate and propionate. In concert with the increased level of TGF-β, *Clostridium* promoted the expression of MMP2, 9 and 13 on the surface of IECs, which rendered latent TGF-β activation in the colon (Atarashi et al., 2011; Bauche and Marie, 2017). Chusri et al. (2016) showed that HCV enhanced ROS induction which then phosphorylated JNK and NF-κB in turn. Consequently, TGF-β expression was up-regulated by activated NF-κB (Chusri et al., 2016). Lin et al. (2010) found that HCV induced ROS production also activated p38 MAPK and p42/44 ERK pathways to phosphorylate NF-κB and upregulate TGF-β.

Secondly, special virus proteins interfere with TGF-β signaling components. HCV core proteins activate TGF-β through inducing thrombospondin-1 in extracellular matrix, which binds to the Leu-Ser-Lys-Leu amino acids sequence and alters conformation of latency associated protein (Benzoubir et al., 2013). HBV-encoded pX oncoprotein and HBV X protein facilitated TGF-β signaling via potentiating nuclear translocation of SMAD4 transcription complex and stabilizing p-SMAD2/3, respectively (Lee et al., 2001; Liu y. et al., 2016). It also activated c-Jun N-terminal kinase/pSMAD3L pathway and inhibited canonical TGFβRI/pSMAD3C to add epithelial proliferation in HCC cell lines (Wu et al., 2016). Additionally, HBV upregulates SMAD7, an inhibitor of TGF-β signaling, leading to resistance of host cells to TGF-β induced apoptosis in HCC (Liu et al., 2015). HPV oncoprotein E7 inhibited binding of SMAD complex to SMAD binding sequence, which attenuated TGF-β signal transduction in E7 expressing cell lines and contributed to tumorigenesis (Lee et al., 2002). Similarly, HPV E5 blocked TGF-β signaling via decreasing SMAD2 phosphorylation and SMAD4 nuclear translocation (French et al., 2013).

**FIGURE 1:**
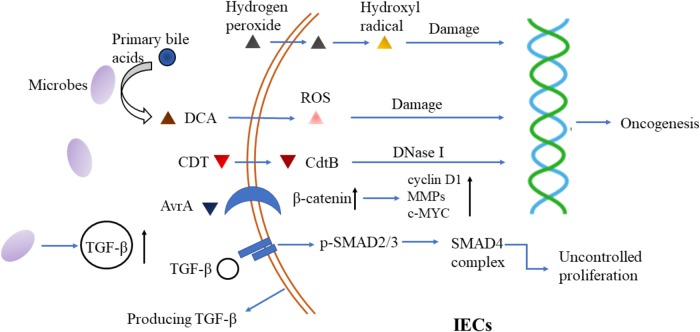
Microbiota-epithelial interaction and its interference by TGF-β signaling. Microbial metabolites (hydroxyl radical and ROS for example) and bacterial derived toxins (such as CDT) induce DNA damage and trigger oncogenesis in epithelial cells. Additionally, bacterial virulence factors, such as AvrA, activate β-catenin signaling to promote proliferation of epithelial cells. Besides, microbes stimulate TGF-β production by IECs through their metabolites and specific proteins, which then triggers uncontrolled proliferation in turn. DCA, deoxycholic acid; ROS, reactive oxygen species; CDT, cytolethal distending toxin; MMP, matrix metalloproteinase; TGF-β, transforming growth factor-β; IECs, intestinal epithelial cells.

Finally, TGF-β influences flora-barrier interaction in association with DCs and regulatory T cells (Tregs). Infection with *Citrobacter rodentium* induces apoptosis of IECs. DCs took part in the phagocytosis of these apoptotic cells and were activated to synthesize TGF-β, IL-6, and IL-23 (Brereton and Blander, 2010). And microbiota induced TGF-β facilitates the expression of fibroblast growth factor 2 (FGF2) in Treg cells. FGF2 cooperates with IL-17 to repair damaged epithelium and control microbes outgrowth, to suppress colitis and colon carcinoma associated with colitis (Song et al., 2015) (**Figure 1**).

## Microbiota-Immune Interaction and Its Interference by TGF-β Signaling

Inflammatory disorders, including pro-inflammatory and anti-inflammatory responses elicited by microbes, may take place when epithelial barrier deteriorate and bacterial translocate. It has generally been accepted that inflammation is a link between microbiota and cancer progression as described by Virchow (1881) in the 19th century. As a prime example, inflammation induced by *H. pylori* with gastritis and gastric ulcers is strongly associated with gastric adenocarcinoma (Marshall and Warren, 1984). Inflammation drives a tumor permissive niche, in which the increased production of cytokines, chemokines and growth factors, as well as nitrogen species and reactive oxygen involve in stimulating cell proliferation and/or inhibiting cell apoptosis to facilitate oncogenesis (Akin and Tozun, 2014; Irrazabal et al., 2014; Zitvogel et al., 2015). Moreover, inflammation may alter microbial composition, increase microbial translocation and promote outgrowth of specific bacteria possibly by production of specific metabolites which allow them to thrive (Arthur et al., 2012; Elinav et al., 2013; Schwabe and Jobin, 2013). Patwa et al. (2011) showed that chronic intestinal inflammation induced stress-response genes expression in gut commensal bacterium, which would confer bacterium environmental adaptability. Bacterial antigens are detected and signaled by the Toll-like receptors (TLR) through NF-κB pathway, which is a key mediator triggering cancer-associated inflammation (Karin and Greten, 2005; DiDonato et al., 2012; Bhatt et al., 2017). In turn, microbes also elicit immunosuppression responses and contribute to tumor-immune evasion. *F. nucleatum* may directly engage TIGIT, a receptor expressed on some T cells and natural killer (NK) cells, to block the antitumor responses of NK cells dependent killing (Gur et al., 2015). Microbial derived butyrate, an inhibitor of histone deacetylase which leads to the activation of forkhead box P3 (FoxP3) regulator and signals through G protein-coupled receptors, could induce naive T cells and dendritic cells to differentiate into Tregs to inhibit anti-tumor immune responses (Gur et al., 2015). These indicated that the association between the microbiota and the host immune system may result in a cause of inflammation and development or progression of cancer.

Increasing evidence showed that TGF-β was an important cytokine in the development of cancer involved in microbiota and immune reaction. Besides of IECs as mentioned above, lamina propria dendritic cells (LPDCs) are also suggested to secrete more TGF-β under stimulation by microbiota-mediated signaling and microbiota-derived products (Bauche and Marie, 2017). In response to *Clostridium butyricum*, LPDCs induced TGF-β1 production through a cooperation of TLR2-AP-1 and TGF-β-SMAD pathway (Kashiwagi et al., 2015). Also, Atarashi et al. (2008) have demonstrated that commensal bacteria derived ATP increased integrin-alphaV and -β8 expression on inflammatory DCs, which activated latent TGF-β (Boucard-Jourdin et al., 2016). TGF-β level in the gut is directly and indirectly modulated by microbes, which impacts the complex interplay with host and microbiota through its modulation of Tregs, Th17 cells and B cells.

### Immune-Dampening Tregs

Tregs, abundant within the lamina propria in colon, suppress anti-tumor effector CD4^+^ and CD8^+^ T cells responses to maintain immune tolerance to tumor antigens and microbial antigens (Bauche and Marie, 2017). TGF-β and other cytokines, to some degree, decided differentiation of CD4^+^ T cells into Tregs or Th17. TGF-β can induce Foxp3 expression in CD25^-^ cells from periphery and convert these cells into CD4^+^CD25^+^ induced Treg (iTreg) cells (Khattar et al., 2009). High concentrations of TGF-β represses IL-23 receptors (IL-23R) expression so that it contributes to Tregs differentiation. TGF-β induces retinoic acid receptor-related orphan receptor γt (RORγt) co-expression in CD4^+^ T cells with Foxp3, which could repress RORγt and result in Tregs differentiation. Whereas, the repression could be removed by IL-6, IL-21, and IL-23 (Zhou et al., 2008).

Microbiota and microbiota derived metabolites are also involved in the process of Foxp3 expression. Commensal *B. fragilis* promotes immunologic tolerance through producing polysaccharide A (PSA), a symbiosis factor. PSA directly stimulates TLR2 on CD4^+^ T cells to strongly enhance the function and expression of Foxp3, IL-10, and TGF-β (Round et al., 2011). Butyrate may directly result in Foxp3 transcription to induce Tregs among CD4^+^ T cells through its inhibition of histone deacetylation in conserved non-coding sequence 1 and promoter regions of the Foxp3 locus (Furusawa et al., 2013). On the other hand, butyrate also indirectly enforces Tregs differentiation by inducing IECs secreting TGF-β (Bauche and Marie, 2017).

### Pro-inflammatory Th17 Cells

The differentiation of Th17 cells, important pro-inflammatory cells in many solid tumors, is stimulated by microbes and is influenced by activated TGF-β under inflammatory conditions within intestines (Veldhoen et al., 2006; Ivanov et al., 2008; Wilke et al., 2011). *Segmented filamentous bacteria* can directly adhere to IECs and induce the production of serum amyloid A proteins 1 and 2, members of acute-phase response proteins family in response to infection or inflammatory, which in turn promote DCs-mediated Th17 cells differentiation in lamina propria and IL-17a secretion (Ivanov et al., 2009; De Simone et al., 2013; Sano et al., 2015). It has been suggested that commensal microbes stimulated CD172α(+) LPDCs inducing Th17 cells partly mediating by TLR5. Once stimulated with flagellin, CD172α(+) LPDCs co-expressed TLR5 can secrete IL-6, IL-23, and TGF-β, which mediate CD4^+^ T cells to develop into Th17 cells, whereas TLR5-deficient LPDCs do not (Liu H. et al., 2016). While it induces Tregs differentiation at a high concentration, TGF-β at low concentrations is in favors of IL-23R expression and promotes Th17 cells differentiation in cooperation with IL-6 and IL-21 (Zhou et al., 2008). With their distinctively secretion of IL-17 that enhances angiogenesis and induces tumor cells proliferation in immune-deficient hosts or patients with already existed chronic inflammation, Th17 cells are negatively correlated with the prognosis of patients with CRC (Numasaki et al., 2005; Tosolini et al., 2011; Pickup et al., 2013; Song et al., 2014; Gagliani et al., 2015).

### IgA^+^ B Cells

Transforming growth factor-β signaling has been reported to regulate responsiveness of B cells to antigens, IgA production and IgA class-switch recombination (CSR) on SMADs-dependent pathways. IgA, a predominant antibody for mucosal surfaces, confers the ability for IgA^+^ B cells to help maintain the integrity of epithelial barrier and modulate bacterial composition within lumen (Klein et al., 2006). *Alcaligenes* which inhabits Peyer’s patches and lymphoid follicles, is shown to contribute to CSR through increasing TGF-β as well as IgA-enhancing cytokine IL-6 production, when cocultured with DCs isolated from Peyer’s patches of WT mice (Obata et al., 2010). *Alcaligenes* has also been reported to bear genes coding for nitric oxide reductase (NOR) which reduces NO. NO is implicated in TGFβRII expression on B cells and regulates TNFα/iNOS-producing DCs mediated IgA CSR (Kukimoto et al., 2000; Tezuka et al., 2007; Bauche and Marie, 2017). In addition, DCs stimulated with microbes induce integrin α4β7 and chemokine receptor 9 (CCR9) to imprint gut-homing specificity on B cells and promote IgA production (Tezuka et al., 2007; Ruane et al., 2016).

**FIGURE 2:**
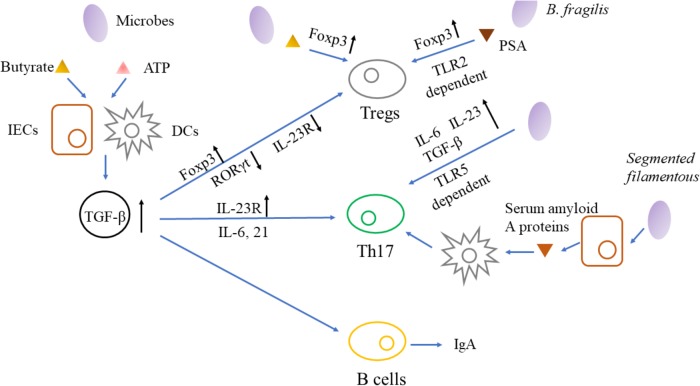
Microbiota-immune interaction and its interference by TGF-β signaling. Bacterial antigens which are detected and signaled by TLRs, could elicit tumor-permissive immune responses by activating Tregs and Th17 cells. In response to microbial metabolites, IECs and DCs secret more TGF-β that impacts the development, proportion and function of Tregs, Th17 cells and B cells, resulting in immune-evasion of tumor cells and regulating the interaction between microbiota and hosts in turn. IECs, intestinal epithelial cells; DCs, dendritic cells; TGF-β, transforming growth factor-β; RORγt, retinoic acid receptor-related orphan receptor γt; IL, Interleukin; Tregs, regulatory T cells; Th17, T helper 17; TLR, Toll-like receptors; PSA, polysaccharide A; IgA, immunoglobulin A.

Collectively, microbiota modulates the production of TGF-β that impacts the development, proportion and function of immune cell subsets, resulting in inflammation and regulating the interaction between microbiota and hosts in turn (**Figure 2**).

## The Mutual Synergy Between Epithelial Barrier Function and Immune Responses

**FIGURE 3:**
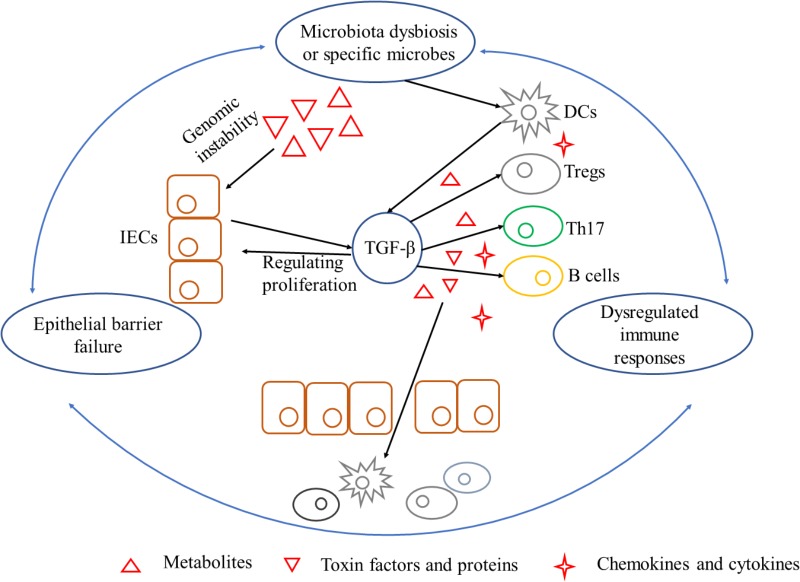
A triangle during the process of carcinogenesis driven by microbiota dysbiosis and its interference with TGF-β. Mechanisms by which microbiota dysbiosis induced carcinogenesis include breaking epithelial barrier, inducing DNA damage in epithelial cells, eliciting inflammation and dampening immune-surveillance of transformed cells. Barrier deterioration is a stimulating factor that triggers locally chronic inflammation to aggravate epithelial barrier failure in turn and exacerbate carcinogenesis. TGF-β, produced by microbes stimulated IECs and inflammatory cells, is a pluripotent cytokine that plays an important role in exacerbating unbalance of the triangle. TGF-β can be tumor-permissive by promoting proliferation and inhibiting apoptosis of epithelial cells. On the other hand, TGF-β is in favor of immune-evasion of tumor antigens elicited by special microbes. IECs, intestinal epithelial cells; TGF-β, transforming growth factor-β; DCs, dendritic cells; Tregs, regulatory T cells; Th17, T helper 17.

As stated, gut flora dysbiosis breached epithelial barrier to provoke immune responses indirectly. As a prime example, fiber-fermenting bacteria is diminished by fiber-free diets but *Akkermansia muciniphila* and *Bacteroides caccae* are increased which degrade mucus in the lumen. Mucus degrading increased the susceptibility of *Citrobacter rodentium* and resulted in “leaky gut” to activate intraepithelial lymphocytes and form chronic inflammation environment (Bhatt et al., 2017). On the other side, locally chronic inflammation is a risk factor for initiating carcinogenesis since epithelial cells transformation accumulates. Leukocytes and other phagocytic cells could generate reactive oxygen and nitrogen species during immune responses, which lead to DNA damage and permanent genomic alterations in epithelial cells (Coussens and Werb, 2002). It is noteworthy that chemokines could also regulate neoplastic cells proliferation and metastasis, as well as angiogenesis of in extracellular matrix (Coussens and Werb, 2002). Carcinogenesis induced by chronic inflammation is also required for signaling pathways such as NF-κB and TGF-β secreted by immune cells as mentioned above (**Figure 3**).

## Conclusion

Colorectal cancer involves in an intricate regulation of tumor cells, microorganism, and non-neoplastic cells (Gagniere et al., 2016). They form an interdependent triangle of microbial dysbiosis, epithelial barrier breach and chronic inflammation in microbiota driven carcinogenesis. And dysregulation of any one side may break the whole equilibrium (Schwabe and Jobin, 2013). Notably, infection of pathogens or commensal bacteria dysbiosis may break epithelial barrier and shape pro-tumorigenic inflammation, leading to cell proliferation. Barrier deterioration, on the one hand, gives microbes and their by-products access to epithelial cells and immune cells (Brennan and Garrett, 2016). On the other hand, perpetual injury triggers locally chronic inflammation and oncogenesis, which then aggravate epithelial barrier failure and microbiota dysbiosis in turn. Bacterial translocation induced inflammation mediates carcinogenesis and inhibits immune-surveillance, as well as contributes to the overgrowing of genotoxic microbes (Arthur et al., 2012). As such, the dysregulation of any one side may break the whole equilibrium, and then forward-amplifying loops formed.

In addition, microbiota functions in anticancer drug metabolism and alters host responses to several drugs. For example, the platinum chemotherapeutic oxaliplatin triggers cancer cell death through forming platinum DNA adducts and intrastrand cross-links. Its efficacy is depended on microbial derived ROS, with augmented intratumoral oxidative stress promoting oxaliplatin associated DNA damage (Iida et al., 2013). Given the crucial effects that microbiota exert on tumor formation and treatment, the differences of microbiota composition, dietary habits, genetic makeup and living environment should be considered when deciding an optimized treatment modality for individuals. It is also called precision medicine treatment involved in precise dosing, symptom management and improved therapeutic responses (Bhatt et al., 2017).

Then, the role of TGF-β signaling pathway is discussed in microbiota driven oncogenesis. Mounting evidence showed that TGF-β was induced by microorganisms, including *Clostridium*, *Bacteroides*, and *Enterobacteriaceae* (Atarashi et al., 2013; Ihara et al., 2017). TGF-β is a pluripotent cytokine that regulates epithelial barrier and immune responses. TGF-β can be tumor-permissive by promoting proliferation and inhibiting apoptosis, but also it is in favor of immune-evasion of tumor antigens elicited by special microbes. In this regard, microbiota increases TGF-β production, as well as reciprocally facilitating the function in oncogenesis. This review indicates that dysregulated microbiota induces carcinogenesis through destabilizing the interdependent interaction of microbiota, epithelial barriers and inflammation, and TGF-β signaling pathway probably plays an important role in exacerbating deviating from homeostasis. As such, it holds great promise for us to look for new therapeutic targets in human cancers relative to microbiota.

## Author Contributions

XP was mainly responsible for the manuscript writing. X-HR, Y-JT, and Q-MC assisted in writing. Y-LT and X-HL provided suggestions on the ideas and performed the final corrections.

## Conflict of Interest Statement

The authors declare that the research was conducted in the absence of any commercial or financial relationships that could be construed as a potential conflict of interest.
